# The Dispensatory of the United States of America

**Published:** 1845-07

**Authors:** 


*The Dispensatory of the United States of America.* By GEORGE B. WOOD, M. D., Professor of Materia Medica and Pharmacy in the University of Pennsylvania; one of the Physicians of the Pennsylvania Hospital, &c. &c.; and FRANKLIN BACHE, M. D., Professor of Chemistry in Jefferson Medical College of Philadelphia; one of the Vice Presidents of the American Philosophical Society, &c. &c. Sixth Edition, carefully Revised. Philadelphia, Grigg & Elliot, 1845.

Of the general excellence of this work it is scarcely necessary to speak. It is so extensively known to the physicians and pharmaciens of this country, and has received such substantial evidences of their approval, as to leave no doubts as to its merits. No other work relating to medicine, in this country, or perhaps in any other, has passed through so many large editions in the same space of time, or received more universal commendation. The natural and commercial history of the various articles of the Materia Medica, their pharmaceutical preparation, doses, modes of administration, chemical properties and incompatibilities, are all set forth with great clearness and accuracy. The pains taken with former editions, and the short period of time which has elapsed since the publication of the last or fifth edition, (less than two years,) have left the authors comparatively little to add in the present. They have been careful, however, to select and condense from the periodical journals, every thing of value which came within the scope of the work, not embraced in the last edition; so that the present may be regarded not only as a full and clear exposition, but as the *latest* account of the various articles and processes to be sought for in a work of this kind.

Another subject of praise is to be found in the mechanical execution of this large volume, which accords well with its contents.

